# Isoliquiritigenin, a Bioactive Blood Component Derived from Licorice, Activates Nrf2 Enzymes to Confer Protection Against Radiation-Induced Nerve Injury

**DOI:** 10.3390/antiox15050614

**Published:** 2026-05-13

**Authors:** Juan Yao, Jiaqi Ma, Huanhuan Lin, Changxin Shao, Xuefeng Liu, Xiaojie Jin

**Affiliations:** 1 College of Pharmacy, Gansu University of Chinese Medicine, Lanzhou 730000, China; 19958587446@163.com (J.M.);; 2 Gansu Phammaceutical Industry Innovation Research Institute, Lanzhou 730000, China

**Keywords:** isoliquiritigenin, Nrf2, Keap1, radiation, oxidative stress

## Abstract

Licorice is a traditional Chinese medicine; however, its bioactive constituents and specific molecular mechanisms responsible for protecting against radiation-induced brain injury remain poorly elucidated. Oxidative stress overactivation acts as the core pathological mechanism underlying radiation-triggered neuronal injury. This study aimed to investigate the neuroprotective effect and underlying mechanism of isoliquiritigenin (ISL), a major blood-absorbed component of licorice, against radiation-induced neural injury in C57BL/6J mice via the Keap1-Nrf2 signaling pathway. Molecular docking and MST analysis verified the strong binding affinity of ISL to Keap1. In vitro, ISL restored the viability of X-ray-irradiated PC12 cells; reduced LDH release and intracellular ROS accumulation; and enhanced SOD1 activity, GSH content, and T-AOC levels. Moreover, ISL upregulated the expression of antioxidant-related genes and induced Nrf2 nuclear translocation. In vivo, oral ISL administration ameliorated radiation-induced cognitive impairment, improved spatial learning and memory, alleviated hippocampal neuronal loss, and increased cerebral cortical Nrf2 expression in C57BL/6J mice. In conclusion, ISL alleviates radiation-induced neuronal injury by suppressing oxidative stress and activating the Keap1-Nrf2 signaling pathway, thus representing a promising therapeutic agent for the prevention and treatment of radiation brain injury.

## 1. Introduction

Radiotherapy is a critical component in the treatment of brain tumors. However, it often causes collateral damage to surrounding healthy neural tissue, resulting in debilitating neurocognitive sequelae. Accumulating clinical evidence demonstrates that both pediatric and adult patients undergoing brain radiotherapy often exhibit progressive cognitive decline. Additionally, exposure to radiation heightens vulnerability to central nervous system symptoms, including neurasthenia, insomnia, and memory deficits, thereby compromising quality of life. Moreover, radiation exposure plays a pivotal role in the onset and progression of neurodegenerative diseases [1,2]. Although amifostine remains the only FDA-approved radioprotector for select radiotherapy indications [3], its clinical utility is severely limited by dose-dependent adverse effects, including hypotension, fever, nausea, and vomiting, even at low doses [4]. This considerably limits its application to counteract injuries caused by high doses of ionizing radiation (IR). Therefore, the development of safer and more efficacious neuroprotective strategies is urgently warranted.

Numerous studies demonstrate that oxidative stress plays a crucial role in the mechanisms of radiation-induced damage [5,6,7]. Radiation causes oxidative stress through reactive oxygen species (ROS) production or the destruction of antioxidant systems [8]. Nuclear factor erythroid-2-related factor 2 (Nrf2) is the key coordinator of cell defense oxidative stress mechanisms, and it modulates the transcriptional activity of antioxidant proteins [9]. Nrf2 is involved in the regulation of the glutathione family, the thioredoxin system, NAD(P)H: quinone oxidoreductase (NQO1), and heme oxygenase 1(HO-1) by modulating the expression of their encoding genes. The cytoprotective effects of Nrf2-directed transcriptional programs enable cells to adapt and survive under stress conditions [10]. Nerve cells exhibit heightened sensitivity to oxidative stress; however, the surgical replacement of dying or dead neurons in neurological disorders involving nerve damage remains highly restricted [11]. Therefore, the activation of Nrf2 exerts a protective effect against radiation-induced oxidative stress, thereby mitigating brain injury and neuronal apoptosis.

Given the limited clinical efficacy of current clinical drugs in treating radiation-induced brain injury, exploring effective preventive and therapeutic agents obtained from natural products may provide a promising approach to address the challenges associated with this condition. Preclinical evidence increasingly supports the notion that certain natural compounds or monomeric antioxidants possess the ability to mitigate radiation-induced damage [7,12]. Licorice is a widely used traditional Chinese medicine (TCM) that exhibits diverse pharmacological effects, such as antioxidant, neuroprotective, anti-inflammatory, antibacterial, antiviral, anticancer, antidepressive, antidiabetic, antiparasitic, antisex hormone, skin effects, anticariogenic, antitussive, and expectorant activities. It has demonstrated potential therapeutic value in the prevention and treatment of various diseases [13]. Research has demonstrated that TCMs containing licorice, along with their bioactive components, can mitigate adverse reactions induced by radiotherapy and enhance the quality of life of patients [14]. The active components in TCM serve as the material foundation for its therapeutic effects, underpinning the pharmacological activities observed in TCM applications. Isoliquiritigenin (ISL) is a bioactive blood component derived from licorice, and it has demonstrated antioxidant, anti-inflammatory, and Nrf2-activating properties in preclinical models; however, whether ISL constitutes the principal bioactive constituent responsible for licorice-mediated protection against radiation-induced neurotoxicity and the molecular mechanisms underlying this potential effect remain incompletely defined and require rigorous mechanistic validation.

In this study, we investigated the neuroprotective effects of ISL, a blood-borne prototype component of licorice, against radiation-induced damage in PC12 cells and brain injury in mice. We found that ISL activates the Keap1-Nrf2-ARE signaling pathway, thereby upregulating phase II detoxifying enzymes and protecting PC12 cells and mice from radiation-induced neurotoxicity. Our findings demonstrate that ISL is a potent small-molecule activator of the Keap1-Nrf2-ARE pathway, suggesting its potential as a candidate drug for mitigating radiation-related oxidative stress disorders. By elucidating the targeting mechanism of ISL on the Keap1-Nrf2-ARE signaling pathway, we provide insights into the molecular basis of its anti-radiation neuroprotective effects and its pharmacological functions in vivo.

## 2. Materials and Methods

### 2.1. Reagents and Chemicals

PC12 cells were purchased from Procell Life Science & Technology Co., Ltd. (Wuhan, China). Licorice was purchased from a pharmacy and originated from Gansu Fuxinghou Biomedical Technology Co., Ltd. (Lanzhou, China). ISL (purity ≥ 98%, purchased from Baoji Chen Guang Biotechnology Co., Ltd., Baoji, China) was dissolved in DMSO and diluted with culture medium or normal saline for cell and animal treatments. 2,2′-Azinobis-(3-ethylbenzothiazoline-6-sulfonic acid) (ABTS) was purchased from Shanghai Fengshou Industrial Co., Ltd. (Shanghai, China). Dulbecco’s Modified Eagle Medium (DMEM) high-glucose culture solution and fetal bovine serum (FBS) were purchased from Thermo Fisher Scientific (Waltham, MA, USA). Pancreatin-EDTA solution was purchased from Shanghai Yuanpei Biotechnology Co., Ltd. (Shanghai, China). A CCK-8 assay kit was purchased from AbMole BioScience (Houston, TX, USA). 2,2-Diphenyl-1-picrylhydrazyl (DPPH), 2,2′-Azobis (2-methylpropionamidine) dihydrochloride (AAPH), a T-AOC assay kit, an SDS-PAGE gel preparation kit, crystal violet, 5× Coomassie Brilliant Blue G-250, and a 4% tissue cell fixative were purchased from Beijing Solarbio Science & Technology Co., Ltd. (Beijing, China). Lactate dehydrogenase (LDH), glutathione (GSH), and superoxide dismutase 1 (SOD1) assay kits were purchased from Nanjing Jiancheng Bioengineering Institute (Nanjing, China). An ROS detection kit, RIPA lysis buffer, phenylmethanesulfonyl fluoride (PMSF), horseradish peroxidase-conjugated goat anti-mouse IgG (H + L), and horseradish peroxidase-conjugated goat anti-rabbit IgG (H + L) were purchased from Shanghai Biyuntian Biotechnology Co., Ltd. (Shanghai, China). Bovine serum albumin (BSA) was purchased from Wuhan Servicebio Technology Co., Ltd. (Wuhan, China). A nuclear and cytoplasmic protein extraction kit, anti-GAPDH rabbit polyclonal antibody, anti-TXNRD1 mouse monoclonal antibody, anti-HMOX1 rabbit monoclonal antibody, and anti-NQO1 rabbit monoclonal antibody were purchased from Sangon Biotech (Shanghai, China) Co., Ltd. Thioredoxin 1 (Trx1) antibody and thioredoxin reductase 1 (TrxR1) antibody were purchased from Cell Signaling Technology, Inc. (Danvers, MA, USA). Nrf2 polyclonal antibody was purchased from GeneAll Signalway Antibody (College Park, MD, USA). RNAiso Plus, a PrimeScript™ RT reagent kit with gDNA eraser, and TB Green^®^ Premix Ex Taq™ II were purchased from Takara Biomedical Technology Co., Ltd. (Beijing, China).

### 2.2. Blood Component Analysis

#### 2.2.1. Sample Preparation

Licorice powder was prepared by grinding licorice slices using a grinder. The licorice powder (0.5 g) was suspended in 4 mL distilled water, refluxed at 100 °C for 1.5 h, cooled to room temperature, and centrifuged at 3000× *g* for 10 min, and the supernatant was collected and stored at −80 °C.

Twelve 8-week-old SPF male C57BL/6J mice were randomly assigned to control or licorice decoction groups (n = 6 per group) and treated by oral gavage. They were maintained in an animal room with an environmental temperature of 22 ± 2 °C and a relative humidity of 55 ± 10%, under a 12 h light/dark cycle, with free access to standard feed and drinking water. The mice in the licorice-containing serum group were given 0.2 mL/10 g licorice decoction by gavage, and the mice in the blank serum group were given the same volume of normal saline by gavage once a day for 3 days. The mice were fasted for 12 h before the last dose. One hour after gavage on the fourth day, blood samples were collected by enucleation of the eyeball. The blood was then centrifuged at 4 °C and 3000 rpm for 10 min to separate the serum. The supernatant was transferred to a clean centrifuge tube, centrifuged again at 4 °C and 12,000 rpm for 10 min, and then stored at −80 °C for later use. This allowed us to obtain the licorice drug-containing serum and the blank serum.

#### 2.2.2. UHPLC-Q-Exactive Orbitrap MS Analysis

Chromatography was performed on an ultra-high-performance liquid chromatograph (Dionex Ultimate 3000) (Thermo Fisher Scientific, Waltham, MA, USA). A Waters ACQUITY UPLC BEH C18 column (2.1 mm × 100 mm, 1.7 μM) (Waters Corporation, Shanghai, China) was used with 0.1% formic acid aqueous (A)–acetonitrile (B) as the mobile phase. The gradient elution sequence was as follows: 0–0.5 min with 5% B, 0.5–1.0 min with 5% B, 1.0–9.0 min with 5–100% B, 9.0–12.0 min with 100% B, and 12–15 min with 5% B. The column temperature was maintained at 35 °C, and the volume flow rate was set at 0.3 mL/min. The injection volume for each sample was 5 μL, and the samples were kept in the autosampler at 4 °C throughout the analysis.

Mass spectrometry analysis was performed using a Q-Exactive mass spectrometer. Mass spectrometry was performed using an electron spray ionization (ESI) source in positive- and negative-ion modes. The ESI source conditions were as follows: nebulizer gas, 60 psi; heater gas, 60 psi; curtain gas, 30 psi; ion source temperature, 320 °C; and ion spray voltage floating (ISVF) ± 3500 V (for both positive and negative modes). The mass spectrometry scanning range was set at 80–1200 Da, with a product ion scan resolution of 17,500. The mass spectrometry scan accumulation time was 0.20 s/spectra, and the product ion scan accumulation time was 0.05 s/spectra. The collision energy was 35 ± 15 eV, and the secondary mass spectrometry declustering potential (DP) was ±60 V (for both positive and negative modes). The data were acquired in information-dependent acquisition (IDA) mode, and the high-sensitivity mode was used. The IDA settings were as follows: isotope exclusion within a 4 Da range and up to 6 candidate ions monitored per cycle.

#### 2.2.3. Data Processing and Analysis of UHPLC-Q-Exactive Orbitrap MS

The data collected were preprocessed using Compound Discoverer 3.0 software. The blood components of licorice were identified using Venn analysis and the PubChem website.

### 2.3. Binding Ability and Molecular Dynamics Simulation of the ISL-Keap1 Interaction

#### 2.3.1. Molecular Docking

Molecular docking was conducted between ISL and Keap1. The Keap1 crystal structure was retrieved from the RCSB PDB database; ligand-binding pocket residues were validated using PyMOL 2.2.0, and non-physiological metal ions were removed to preserve structural fidelity for docking simulations. The Keap1 structure (PDB: 4L7B) was preprocessed using the Protein Preparation Wizard module in Schrödinger 2015, including removal of water molecules, addition of missing hydrogen atoms, optimization of bond orders, creation of disulfide bonds, and restricted energy minimization. The structure was minimized using the OPLS_2005 force field. Structural files of ISL were retrieved from the PubChem database and imported into Schrödinger 2015. The LigPrep module was employed for small-molecule structure optimization and conformation generation. In LigPrep, the OPLS_2005 force field was selected, the pH was controlled at 7.0 ± 2.0, and the Epik algorithm was used to generate the protonation states and corresponding low-energy conformations of the small molecules for subsequent docking. Subsequently, the Receptor Grid Generation tool in the Glide module was used to define the docking site of ISL and Keap1 using the native ligand of Keap1, and the active pocket was generated as the docking grid. Then, ligand docking in the Glide module was carried out in the SP (standard precision) mode.

#### 2.3.2. Molecular Dynamics (MD) Simulation

Molecular dynamics (MD) simulations were performed for the Keap1–ISL systems using the pmemd module of Amber20. Structural optimization and electrostatic potential calculations for the small-molecule ligands were carried out using Gaussian 09 at the HF/6-31G* basis set level. Partial atomic charges of the ligands were subsequently fitted using the RESP [15,16] (restrained electrostatic potential) method via the Antechamber module in the Amber Tools package. Force-field parameters for the small-molecule ligands were generated with the GAFF [17] (General Amber Force Field), while the Keap1 protein was described using the ff14SB [18] force field. Each complex system was solvated in a periodic water box constructed with the TIP3P [15] water model, with a minimum distance of 12 Å between the solute surface and the edge of the water box. An appropriate number of Na^+^ counterions was added to maintain the electroneutrality of the solution. Topology and coordinate files for MD simulations were generated using the tleap module.

Prior to performing 100 ns simulations, the system was subjected to multiple steps of energy minimization and equilibration. First, harmonic restraints of 10.0 kcal/(mol·Å^2^) were applied to the heavy atoms of the protein and ligand, and the steepest descent and conjugate gradient methods were used to minimize the energy of water molecules and ions. Subsequently, all restraints were removed, and unrestrained energy minimization was performed on the entire system to eliminate unreasonable steric clashes. Next, under the NVT ensemble, the system was gradually heated from 0 K to 300 K within 20 ps, with temperature controlled by the Langevin thermostat to ensure a uniform temperature distribution, providing reasonable initial conditions for the subsequent equilibration stage. Afterwards, 50 ps of equilibration was conducted under the NPT ensemble, with harmonic restraints of 5.0 kcal/(mol·Å^2^) imposed on the backbone atoms of the protein. The Berendsen pressure coupling method was used to maintain the system pressure at 1 atm, allowing the system to reach preliminary equilibrium under isothermal–isobaric conditions. After completion of equilibration, three independent unrestrained conventional MD simulations of 100 ns each were performed for each complex system at 300 K and 1 atm to ensure the reproducibility of the results. The simulation time step was set to 2 fs, and the SHAKE algorithm was employed to constrain the bond lengths of all hydrogen-containing atoms to improve computational efficiency. The positional coordinates and energy information of the system were saved every 10 ps for subsequent analysis.

#### 2.3.3. Binding Free Energy

Trajectory analyses were performed using the CPPTRAJ [19] module in Amber 20. To eliminate interference from highly flexible regions in the Keap1 protein, root-mean-square deviation (RMSD) and root-mean-square fluctuation (RMSF) were calculated after aligning trajectories to the backbone atoms of the Kelch domain. The binding free energy between the ligand and the receptor was computed using the Molecular Mechanics/Generalized Born Surface Area (MM/GBSA) [20] approach. Calculations were carried out over the last stable 10 ns from three independent molecular dynamics simulations via the MMPBSA.py [21] module, and the final binding free energy and its individual energy components were presented as the mean ± standard deviation (mean ± SD). The free energy components included the electrostatic interaction contribution (Δ*E_ele_*), van der Waals interaction contribution (Δ*E_vdw_*), and solvation energy contribution (Δ*G_solv_*). The solvation free energy consisted of nonpolar (Δ*G_nonpol_*) and polar (Δ*G_pol_*) contributions. The calculation formula of the MM/GBSA method is as follows:

(1) $$∆G_{\mathrm{bind}}\;=\;∆E_{\mathrm{vdw}}\;+\;∆E_{\mathrm{ele}}\;+\;∆G_{\mathrm{pol}}\;+\;∆G_{\mathrm{nonpol}}$$

(2) $$∆G_{\mathrm{gas}}=∆E_{\mathrm{vdw}}+∆E_{\mathrm{ele}}+∆E_{\mathrm{int}}$$

(3) $$∆G_{\mathrm{solv}}=∆G_{\mathrm{pol}}+∆G_{\mathrm{nonpol}}$$

(4) $$∆G_{\mathrm{calc}}^{\text{per-resdiue}}=∆E_{\mathrm{vdW}}^{\text{per-resdiue}\;}+∆E_{\mathrm{ele}}^{\text{per-resdiue}}+∆G_{\mathrm{pol}}^{\text{per-resdiue}}+∆G_{\mathrm{nonpol}}^{\text{per-resdiue}}$$

#### 2.3.4. Microscale Thermophoresis (MST)

The Keap1 protein was labeled with RED-NHS dye. ISL was serially diluted in Assay Buffer containing 5% DMSO (concentration range: 0.763 nM to 25 µM), and then mixed with an equal volume of labeled protein (final concentration: 0.5–1 µM). The mixture was incubated at room temperature for 15 min before being loaded onto the MST instrument (Monolith NT.115, Nano Temper Technologies GmbH, Munich, Germany). Detection conditions were as follows: excitation power 20%, MST power 40%, and temperature 25 °C. The dissociation constant (Kd) was fitted using MO Affinity Analysis v2.3 software. Data from at least three independent replicate experiments were analyzed, and the results are presented as the mean ± SEM.

### 2.4. In Vitro Free Radical Scavenging Activity

The different concentrations of sample solutions (100 μL) were mixed with DPPH solution (900 μL). The mixtures were vortexed thoroughly and incubated at room temperature for 30 min in the dark, after which the absorbance was determined spectrophotometrically at 517 nm. The ABTS working solution was prepared by mixing ABTS solution with potassium persulfate solution and allowing the mixture to stand in the dark for 24 h. Subsequently, the solution was diluted with absolute ethanol at room temperature until its absorbance reached 0.7 ± 0.02 at 734 nm. Afterwards, ABTS working solution (900 μL) was blended with sample solution (100 μL), and the absorbance at 734 nm was measured following a 30 min incubation.

Erythrocytes (RBCs) were obtained from fresh sheep blood by centrifugation and suspended in PBS to prepare a 10% RBC suspension at a ratio of 1:9 (RBCs:PBS). After preparing different reaction systems, the mixtures were pre-incubated in a water bath at 37 °C for 5 min, and hemolysis was subsequently initiated by the addition of AAPH solution. The reaction mixtures were shaken periodically to maintain RBCs in a suspended state. Aliquots were collected at 30 min intervals, diluted with 0.9 mL of NaCl solution, and centrifuged at 3000 rpm for 5 min to pellet the erythrocytes. The hemolysis rate was calculated based on the absorbance of the supernatant measured at 540 nm.

### 2.5. Cell Culture

The PC12 cells were maintained in a humidified 5% CO_2_ incubator at 37 °C. The growth medium consisted of high-glucose Dulbecco’s Modified Eagle Medium (DMEM), supplemented with 10% fetal bovine serum (FBS) and 100 U/mL penicillin–streptomycin.

### 2.6. Cell Viability

PC12 cells were plated in 96-well plates at a seeding density of 5000 cells per well and allowed to attach overnight. Subsequently, the cells were exposed to ISL at final concentrations of 1, 2, or 4 μM for 24 h. After treatment, 10 μL of CCK-8 reagent was added to each well, followed by a 2 h incubation at 37 °C. Absorbance was then quantified at 450 nm using a microplate spectrophotometer. For the X-ray radiation injury model, the PC12 cells were cultured in complete medium until they reached 70% confluency and fully adhered to the substrate. The cells were then exposed to IR using an X-ray irradiator (Model No:X-RAD225, Cabinet kV:225 kV, Serial No:1511-2761-02, FDA Accession No:1010288-000, Input Voltage:1PH 230VAC, 50/60 HZ, FLA:40 Amp) at doses of 4 Gy. The cells were then returned to the incubator for 24 h before further analysis. Furthermore, to investigate whether the protective effect of ISL against radiation-induced PC12 cell injury is dependent on the Nrf2 pathway, cells were seeded in 96-well plates and randomly divided into 6 groups: a control group, model group, positive drug tBHQ group (20 μM), ISL (4 μM) group, tBHQ + ML385 (20 μM) group, and ISL combined with ML385 (20 μM) group. After drug administration and radiation treatment, 10 μL of CCK8 solution was added to each well. Following incubation for 2 h, the absorbance was measured at a wavelength of 450 nm using a microplate reader.

### 2.7. Measurement of LDH, T-AOC, GSH, SOD1, HO-1, NQO1, Trx1, and TrxR1

PC12 cells were plated in 60 mm culture dishes and allowed to adhere overnight. Thereafter, the medium was replaced with fresh complete medium supplemented with ISL at final concentrations of 1, 2, or 4 μM. The cells were then irradiated with a single dose of 4 Gy X-ray. Following a 24 h post-irradiation incubation, the levels of LDH, T-AOC, GSH, SOD1, HO-1, NQO1, Trx1, and TrxR1 in the cells were quantified using commercially available assay kits, strictly following the manufacturers’ protocols.

### 2.8. Quantitative Real-Time Polymerase Chain Reaction (qRT-PCR)

PC12 cells were seeded in 60 mm culture dishes and allowed to attach overnight. Subsequently, the cells were treated with 4 μM ISL for 24 h. Total RNA was isolated using the RNAiso Plus reagent (Takara Biomedical Technology Co., Ltd. Beijing, China) following the manufacturer’s protocol. RNA concentration and purity (A260/A280 ratio) were determined using a NanoDrop microvolume spectrophotometer (Thermo Fisher Scientific, Waltham, USA). First-strand cDNA was synthesized from 1 μg of total RNA using a PrimeScript™ RT Reagent Kit (Takara Bio), and qRT-PCR was performed on a CFX96 Touch™ Real-Time PCR Detection System (Bio-Rad, Shanghai, China) with TB Green^®^ Premix Ex Taq™ II (Takara Bio). *GAPDH* served as the endogenous reference gene. Gene-specific primers (listed in Table 1) were designed based on published mRNA sequences and commercially synthesized by Takara Biomedical Technology Co., Ltd. (Beijing, China). Relative mRNA expression levels were calculated using the 2^−ΔΔCT^ method.

### 2.9. Western Blot Analysis

PC12 cells were seeded in 60 mm dishes and treated with ISL for 24 h after overnight attachment. Depending on the experimental objective, whole-cell, cytoplasmic, or nuclear protein fractions were isolated. For total protein extraction, the cells were washed thrice with ice-cold phosphate-buffered saline (PBS) and lysed on ice for 30 min in RIPA lysis buffer supplemented with protease and phosphatase inhibitors. Lysates were centrifuged at 12,500× *g* for 15 min at 4 °C. The supernatants were collected, quantified, and then used immediately or stored at −20 °C for subsequent experiments. Subcellular fractionation into cytoplasmic and nuclear extracts was carried out using a commercially available nuclear/cytoplasmic extraction kit (Beyotime, Shanghai, China), strictly adhering to the manufacturer’s instructions. Protein concentration was determined using the Coomassie Brilliant Blue G-250 dye-binding assay.

For Western blotting, equal amounts of protein were resolved by SDS–polyacrylamide gel electrophoresis (SDS-PAGE) and electrotransferred onto polyvinylidene difluoride (PVDF) membranes. The membranes were blocked for 2 h at room temperature with 2.5% nonfat dry milk in TBST (Tris-buffered saline containing 0.1% Tween-20) and then incubated overnight at 4 °C with primary antibodies diluted in blocking buffer. After three 10 min washes in TBST, the membranes were incubated for 1 h at room temperature with appropriate HRP-conjugated secondary antibodies. Immunoreactive bands were visualized using an enhanced chemiluminescence (ECL) detection system (Thermo Fisher Scientific) and captured digitally using a ChemiDoc™ MP Imaging System (Bio-Rad, Hercules, CA, USA).

### 2.10. Fluorescence Assay

Intracellular ROS Detection: PC12 cells were seeded in 6-well plates, allowed to adhere overnight, pretreated with ISL, and irradiated with a single 4 Gy X-ray dose. After a 6 h post-irradiation incubation, the culture medium was replaced with 1 mL of serum-free DMEM containing 10 μM DCFH-DA per well. The plates were incubated in the dark at 37 °C for 30 min to allow intracellular esterase-mediated conversion of DCFH-DA into fluorescent DCF. After removal of the unbound probe, the cells were rinsed twice with prechilled PBS. Finally, 2 mL of PBS was added to each well, and fluorescence intensity was visualized and recorded using a fluorescence microscope.

Immunofluorescence Analysis of Nrf2 Content and Distribution: PC12 cells were cultured in 35 mm dishes, allowed to attach, and then treated with ISL prior to 4 Gy irradiation. At the designated time point post-treatment, the cells were rinsed twice with PBS, fixed with 4% paraformaldehyde for 30 min at room temperature, and permeabilized with 0.1% Triton X-100 for 10 min. To minimize non-specific background, samples were blocked with 1% bovine serum albumin (BSA) in PBS for 1 h at room temperature. Primary antibody against Nrf2 was applied overnight at 4 °C. After three PBS washes, the cells were incubated with secondary antibodies for 2 h in the dark. Nuclei were counterstained with DAPI for 5 min. Fluorescence images were acquired using a fluorescence microscope with appropriate filters.

### 2.11. Assessment of Cell Apoptosis

PC12 cells were plated in 6-well plates at a density of 1 × 10^5^ cells per well and maintained under standard culture conditions until complete adherence was achieved. The cells designated for the radiation injury model were then exposed to a single 4 Gy dose of X-ray irradiation. Immediately following irradiation, ISL was added at varying concentrations, and the cultures were incubated for an additional 24 h prior to collection. The collected cells were gently resuspended in ice-cold 1× PBS, centrifuged, and washed once to remove residual medium and debris. The resulting pellet was resuspended in 300 μL of 1× Binding Buffer. Aliquots of 100 μL were transferred into separate flow cytometry tubes. To each tube, 5 μL of Annexin V–FITC conjugate and 5 μL of propidium iodide (PI) were added in sequence, followed by careful vortexing or pipetting to ensure uniform staining. The samples were then incubated for 15 min at room temperature in the dark. Finally, 200 μL of 1× Binding Buffer was added to each tube to bring the total volume to 300 μL before flow cytometric analysis.

### 2.12. Animals

All animal experiments were conducted in accordance with the institutional guidelines and received prior approval from the Animal Ethics Committee of Gansu University of Chinese Medicine (Approval No.: SY2024-314). Thirty-six SPF male C57BL/6J mice, weighing 20 ± 2 g, were obtained from Beijing Huafukang Biotechnology Co., Ltd. (Beijing, China). Upon arrival, the animals were acclimatized for one week under controlled SPF conditions—maintained at 22–24 °C, 50–60% humidity, and a 12 h light/dark cycle—in the Animal Experimental Center of Gansu University of Chinese Medicine. After acclimatization, the mice were randomly assigned to either the control group (n = 12) or the radiation injury model group (n = 24). The mice were anesthetized via an intraperitoneal injection of 3% pentobarbital sodium at a volume of 0.2 mL per 10 g body weight. Following anesthesia, the brain region of the mice was exposed to 15 Gy of X-ray at a dose rate of 2 Gy/min to establish a radiation-induced nerve injury model. The mice were placed in a prone position, with the irradiation field restricted to the area between the right ear and the dorsal midline; the rest of the body was protected with lead shielding, and the source–skin distance was maintained at 48 cm [22,23]. Following irradiation, ISL was administered daily via gavage at a dose of 50 mg/kg for 7 consecutive days, after which samples were collected for further analysis [24,25].

### 2.13. HE Staining and Nissl Staining

Brain tissue was collected and promptly washed with pre-cooled PBS, followed by fixation in 4% paraformaldehyde for 24 h. Subsequently, paraffin-embedded sections were prepared and stained with HE or Nissl staining to evaluate the morphological features of the brain tissue.

### 2.14. Morris Water Maze

The learning and memory abilities of the mice were assessed using the Morris water maze two weeks post-radiation. The water maze apparatus was divided into four quadrants, each marked with a distinct color on the pool walls, and the platform was positioned at the center of one quadrant. The pool was filled with water, maintaining the water surface 2 cm above the platform. The water temperature was kept at 24 ± 2 °C, while the laboratory temperature was maintained at 20 ± 2 °C. Titanium dioxide powder was added to the water and stirred thoroughly to obscure the hidden platform from the mice. A camera mounted directly above the maze recorded mouse movements, and water maze video tracking software was used for data recording and analysis. The Morris water maze experiment consisted of two main phases: (1) Positioning navigation: Escape latency (time to reach the platform) and swim paths were recorded over four 120 s trials per day for four days. The mice entered from different quadrants in a randomized order. If the platform was not found within 120 s, latency was capped at 120 s, and the mice were guided to it for a 20 s rest. After each trial, the mice were dried and returned to their cages. (2) Spatial probe test: Twenty-four hours after navigation training, the platform was removed. The mice were released from the quadrant opposite the former platform location, and the number of crossings of the target zone and the time spent in the target quadrant were recorded over 120 s to assess spatial memory.

### 2.15. Statistical Analysis

Statistical analyses were conducted and graphs were constructed using GraphPad Prism 8.0.2. Data are presented as the mean ± standard deviation (SD). Differences between two groups were assessed with Student’s *t*-test, whereas differences among multiple groups were evaluated by a one-way ANOVA. A *p*-value < 0.05 was considered statistically significant.

## 3. Results

### 3.1. UHPLC-Q-Exactive Orbitrap MS Chromatograms

**Figure 1:**
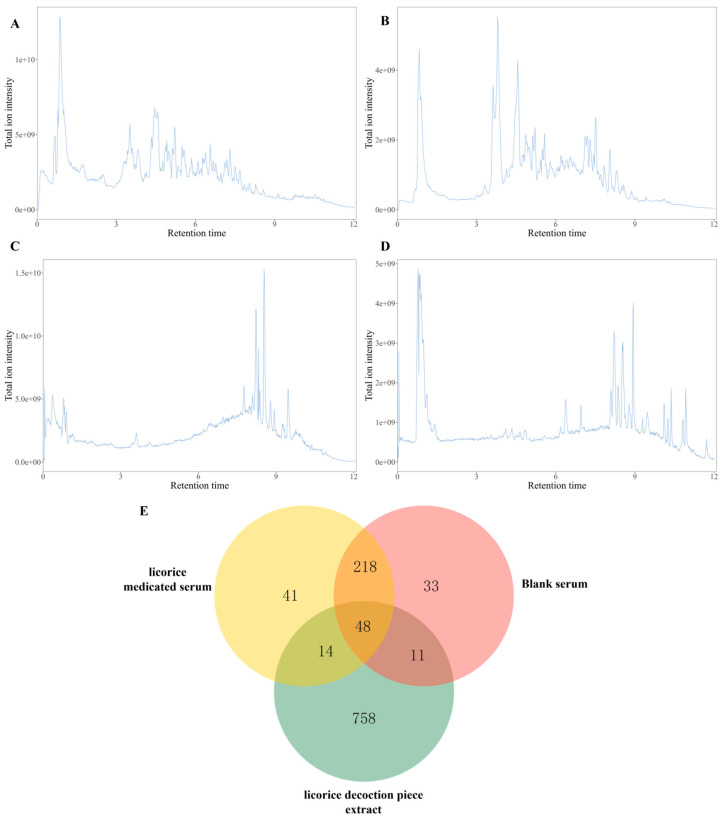
Representative UHPLC-Q-Exactive Orbitrap-MS base peak ion flow chromatograms. (**A**,**B**) Licorice sample chromatograms in positive- (**A**) and negative-ion (**B**) modes. (**C**,**D**) Drug-containing serum samples from licorice-treated mice in positive- (**C**) and negative-ion (**D**) modes. (**E**) Venn diagram analysis of differential components among licorice extract, licorice drug-containing serum, and blank serum.

According to the chromatographic conditions, total ion current (TIC) chromatograms were acquired for the licorice decoction extract (Figure 1A,B) and the corresponding licorice-dosed mouse serum (Figure 1C,D) in both positive and negative ionization modes. A comparative inspection revealed consistent discrepancies in the overall metabolite profiles between the two matrices under each polarity, indicating that licorice constituents underwent substantial biotransformation upon entering the murine system and that the serum metabolome was correspondingly altered after oral administration of the licorice decoction. Figure 1E presents a Venn analysis of the licorice decoction extract, licorice-dosed serum, and blank serum, further delineating the distribution of shared and unique features among these groups.

### 3.2. Binding Ability and Molecular Dynamics Simulation of ISL-Keap1

The in vivo-absorbed constituents and metabolites of licorice were annotated by querying their accurate mass data against the PubChem database, supplemented with literature evidence. This process led to the identification of fourteen licorice-derived xenobiotics in serum, including ISL and 18-β-glycyrrhetinic acid (Table 2). Using molecular docking methods, the binding activity of the core blood-entry components of licorice to the key target Keap1 was evaluated to screen for potential active compounds in licorice. A docking binding affinity threshold of ≤−5.0 kcal/mol was applied to prioritize compounds with energetically favorable interactions with Keap1, resulting in the identification of 11 candidate compounds, including apigenin, naringenin, and ISL. The scoring results are presented in Table 3. ISL is present at a relatively high content in licorice and shows relative specificity in licorice compared with apigenin and naringenin. Therefore, ISL was selected as the main research object in subsequent studies.

**Figure 2:**
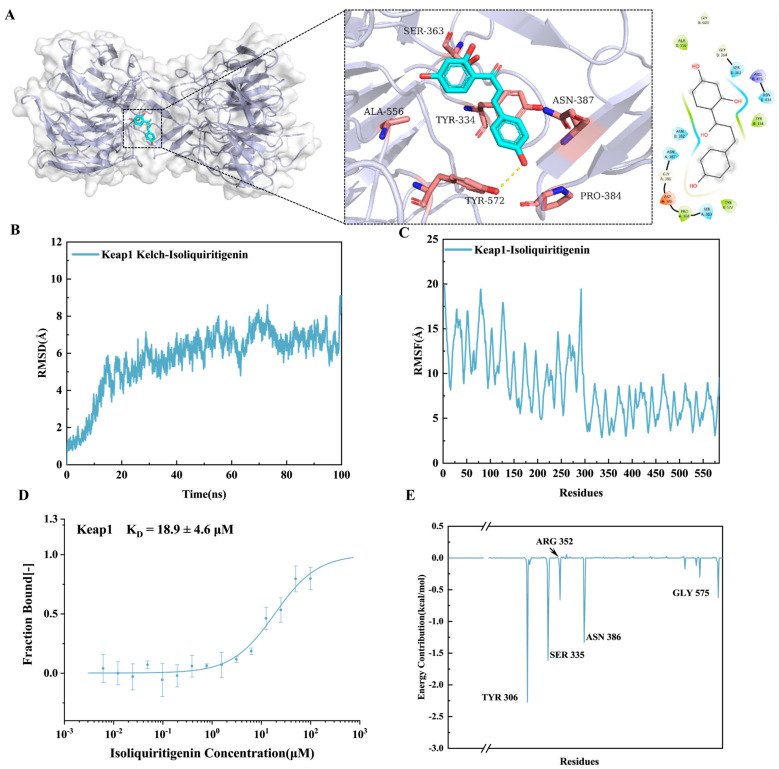
Molecular docking binding mode, MST analysis, and molecular dynamics simulation of the ISL–Keap1 complex. (**A**) Docking binding mode of ISL-Keap1 (including the overall binding diagram of the complex; 3D schematic diagram of the Keap1 dimer and small molecules in the binding pocket; and 2D interaction diagram, in which key amino acids are labeled, in the 3D schematic diagram, the yellow dotted lines represent hydrogen bonds, while the rest are hydrophobic interaction residues and polar residues. In the 2D interaction map, the black solid lines indicate hydrogen bond interactions connecting amino acid residues; green/yellow ellipses represent hydrophobic or van der Waals contact residues, such as TYR-334; blue/orange/purple ellipses respectively represent polar, acidic or basic residues, participating in polar or electrostatic interactions). (**B**) Time-dependent changes in RMSD during the simulation. (**C**) RMSF of amino acid residues during the simulation. (**D**) MST was employed to determine the interaction between ISL and Keap1 (Kd value). (**E**) Residual energy decomposition diagram of ISL-Keap1.

The binding energy of ISL to Keap1 was −5.93 kcal/mol, and the corresponding binding mode is presented in Figure 2A. ISL embeds deeply into the central hydrophobic cavity of Keap1. Keap1 typically exists as a homodimer (as shown in the left panel of Figure 2A), and ISL occupies the canonical Nrf2-binding site. ISL adopts an extended V-shaped or slightly bent conformation that fits well into the shape of the binding pocket. A phenolic hydroxyl group at one end of ISL forms a hydrogen bond with TYR-572, while its benzene ring engages in hydrophobic interactions with TYR-334 and ALA-556 of the Keap1 B chain. Multiple polar residues (such as ASN, SER, and ARG) surround the binding pocket, creating a complementary electrostatic environment with the multiple hydroxyl and carbonyl groups on ISL. As a result, ISL anchors within the pocket via hydrogen bonds formed at both ends and further stabilizes its conformation through hydrophobic interactions between its central backbone and residues, including TYR-334, thereby effectively occupying the binding site. Consequently, ISL effectively occupies the functional binding site and theoretically enables competitive inhibition of the Keap1–Nrf2 interaction.

To evaluate the structural stability of ISL upon binding to Keap1 and to eliminate interference from the inherently highly flexible N-terminal region of the Keap1 protein, 100 ns molecular dynamics simulations were performed on the Keap1–ISL complex system. The conformational adaptation process of the complexes was monitored by calculating the root-mean-square deviation (RMSD) of the relatively rigid Kelch domain. During the simulation, the Keap1–ISL complex underwent prominent conformational changes at the initial stage with a continuous rise in RMSD, and it achieved dynamic equilibrium at 40–100 ns in the mid-to-late simulation period (Figure 2B). The emergence of such equilibria indicates that ISL can induce conformational rearrangements of the Kelch domain and form stable binding modes under different conformational changes. To further explore the local flexibility of individual Keap1 residues, we calculated the root-mean-square fluctuation (RMSF) of the full-length protein. The RMSF curves of amino acid residues in the Keap1-ISL complex system are depicted in Figure 2C. The N-terminal region (residues 1–300) exhibits extremely high flexibility, with a maximum value of 15–20 Å, which accounts for the substantial conformational deviation of the full-length protein in conventional analyses. In contrast, the Kelch domain (approximately residues 300–600), which contains the ligand-binding pocket, shows relatively low and regular fluctuations. This provides a structural basis for the stable binding of ligands. The results from simulations demonstrate that the binding of ISL does not disrupt the overall secondary structural stability of the Kelch domain, thereby enabling a relatively stable interaction with Keap1. In addition, the binding affinity (Kd) of ISL to Keap1 was measured to be 18.9 ± 4.6 µM by an MST assay, further confirming their direct interaction (Figure 2D). In conclusion, ISL can directly and specifically bind to the Keap1 protein.

The binding affinities of ISL–Keap1 were further evaluated by calculating the binding free energies of the complexes using the MM/GBSA method, and the results are presented in Figure 2E and Table 4. The binding free energy of ISL–Keap1 was determined to be −20.65 ± 0.24 kcal/mol. Further decomposition of the energy terms revealed that all energy terms of the ISL–Keap1 system contributed favorably to the binding process. When ISL binds to Keap1, the TYR306, SER335, ARG352, ASN386, and GLY575 residues on Keap1 primarily contribute to the binding energy.

### 3.3. Scavenging of Free Radicals In Vitro

**Figure 3:**
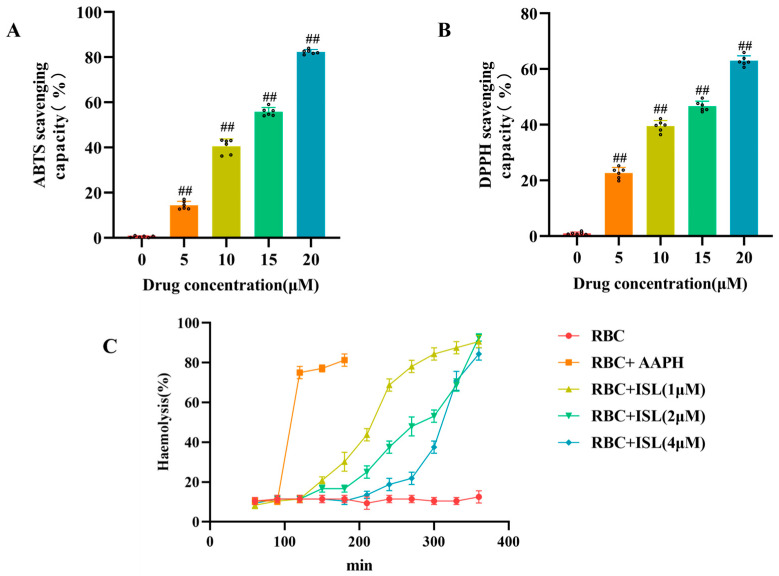
Antioxidant activity of ISL in vitro. (**A**) Measured ABTS and (**B**) DPPH scavenging abilities (n = 6). (**C**) The hemolytic activity of ISL against red blood cells. ^##^ *p* < 0.01 vs. the control group.

Previous investigations have revealed the pharmacological antioxidant activity of ISL [26]. In the present study, we further evaluated the in vitro free radical scavenging potential of ISL. As shown in Figure 3A,B, ISL can effectively scavenge DPPH and ABTS free radicals within the concentration range of 0–20 μM, and the scavenging of DPPH and ABTS free radicals by ISL is dose-dependent. We also found that, in the AAPH-induced intact sheep RBC hemolysis model, ISL can delay the rupture time of red blood cells to varying degrees, protect the integrity of the red blood cell membrane, and alleviate hemolysis of red blood cells. The maximal effect was observed at a concentration of 4 μM, with a marked concentration-dependent trend (Figure 3C).

### 3.4. Protective Effect of ISL on IR-Injured PC12 Cells via Regulation of ROS Accumulation and Apoptosis

**Figure 4:**
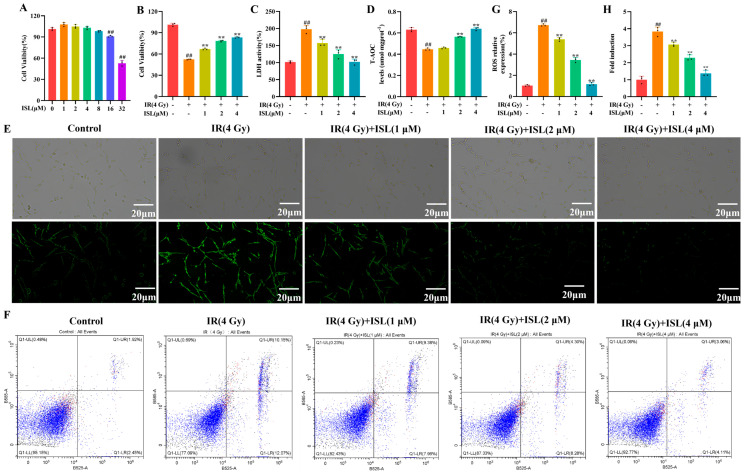
ISL ameliorates radiation-induced damage, ROS accumulation, and apoptosis in PC12 cells. (**A**) Effect of ISL on the viability of PC12 cells. (**B**) Effect of ISL on the viability of radiation-damaged PC12 cells. (**C**,**D**) The effect of ISL on the levels of LDH and T-AOC in PC12 cells after radiation damage. (**E**,**G**) Cellular ROS levels were determined using DCFH-DA (scale bars: 20 μm). (**F**,**H**) Cell apoptosis was detected and quantified via Annexin V-FITC/PI double-staining assay (n = 3). ^##^ *p* < 0.01 vs. the control group; ** *p* < 0.01 vs. the IR group.

The CCK-8 assay was employed to assess the cytotoxicity of ISL against PC12 cells. As shown in Figure 4A, B, ISL had no significant effect on cell viability at concentrations of 1, 2, and 4 μM. Based on these results, the ISL intervention concentrations selected for subsequent experiments were 1, 2, and 4 μM. LDH is a stable cytoplasmic enzyme widely present in various organisms. It is retained intracellularly under physiological conditions but leaks into the extracellular space upon cell injury or death, rendering it a reliable biomarker for plasma membrane integrity. To further verify the protective effect of ISL against IR-induced neurotoxicity, we detected LDH levels in the cell culture supernatant. As illustrated in Figure 4C, the LDH content in the culture medium was markedly elevated following IR exposure relative to that in the control group (*p* < 0.01). Compared with the model group, intervention with different concentrations of ISL markedly decreased LDH release in a concentration-dependent manner (*p* < 0.01). As presented in Figure 4D, we detected the levels of T-AOC in radiation-injured PC12 cells. Compared with the control group, T-AOC activity was significantly downregulated in the model group (*p* < 0.01). By contrast, ISL treatment effectively elevated cellular T-AOC levels in a concentration-dependent fashion, with the maximum protective effect observed at 4 μM (*p* < 0.01).

Additionally, we further detected intracellular ROS changes in irradiated PC12 cells via fluorescence microscopy. As shown in Figure 4E, G, both green fluorescence intensity and intracellular ROS levels were significantly upregulated in the IR group relative to the control group (*p* < 0.01). After ISL treatment, green fluorescence intensity and ROS production were reduced in a dose-dependent manner, with the optimal effect observed at 4 μM ISL (*p* < 0.01). Radiation exposure not only promotes intracellular ROS accumulation but also triggers cell apoptosis. An Annexin V-FITC/PI double-staining assay was further applied to explore the protective role of ISL against radiation-induced apoptosis in PC12 cells. As illustrated in Figure 4F,H, the apoptotic rate of PC12 cells was markedly elevated in the radiation group relative to that in the control group (*p* < 0.01). Notably, ISL intervention remarkably alleviated cellular apoptosis (*p* < 0.01). Collectively, these findings indicate that ISL can effectively suppress radiation-induced apoptosis, thereby exerting a prominent neuroprotective effect.

### 3.5. Effects of ISL on the Antioxidant Enzymes in PC12 Cells

**Figure 5:**
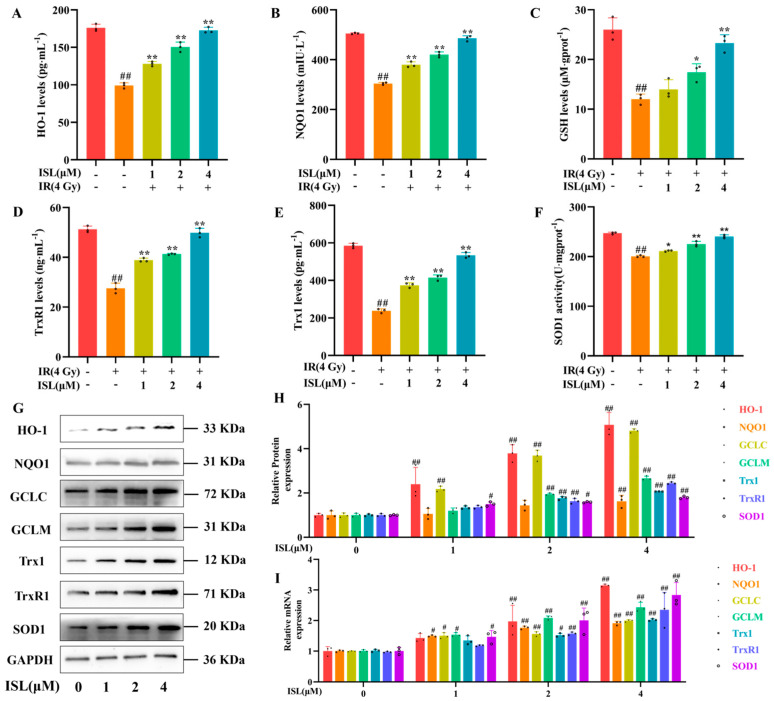
Upregulation of antioxidant enzymes by ISL in PC12 cells. Induction of (**A**) HO-1, (**B**) NQO1, (**C**) GSH, (**D**) TrxR1, (**E**) Trx1, and (**F**) SOD1 activities by ISL in PC12 cells. (**G**) Induction of HO-1, NQO1, Trx1, TrxR1, GCLC, GCLM, and SOD1 protein expression by ISL determined by Western blot. (**H**) Quantification of the band intensity in (**G**). (**I**) Effect of ISL on *HO-1*, *NQO1*, *GCLC*, *GCLM*, *Trx1*, *TrxR1*, and *SOD1* mRNA levels in PC12 cells (n = 3). ^#^ *p* < 0.05, ^##^ *p* < 0.01 vs. the control group; * *p* < 0.05, ** *p* < 0.01 vs. the IR group.

Previous evidence has demonstrated that ISL has a protective effect on nerve injury, and radiation-induced nerve injury is associated with oxidative stress [27]. In the present study, the activities of HO-1, NQO1, GSH, Trx1, TrxR1, and SOD1 were determined to investigate the regulatory effects of ISL on antioxidant enzymes in irradiated PC12 cells. As shown in Figure 5A–F, compared with the control group, the levels of HO-1, NQO1, GSH, Trx1, TrxR1, and SOD1 in the model group were significantly decreased (*p* < 0.01). By contrast, ISL treatment effectively upregulated the expression or activities of HO-1, NQO1, GSH, Trx1, TrxR1, and SOD1 compared with those in the model group (*p* < 0.01). Overall, ISL markedly enhances the activities of antioxidant enzymes in radiation-injured PC12 cells in a dose-dependent manner.

Western blot was performed to detect the protein expression levels of HO-1, NQO1, GCLC, GCLM, Trx1, TrxR1, and SOD1 in each group. As shown in Figure 5G,H, ISL upregulated the expression of these proteins in a dose-dependent manner (*p* < 0.01). Furthermore, qRT-PCR was applied to explore the regulatory effects of ISL on the transcription of antioxidant-related genes. As shown in Figure 5I, ISL treatment markedly upregulated the mRNA expression levels of *HO-1*, *NQO1*, *GCLC*, *GCLM*, *Trx1*, *TrxR1*, and *SOD1* in PC12 cells, with the maximum effect observed at 4 μM (*p* < 0.01). In summary, ISL enhances the expression of antioxidant-related proteins and genes, consequently strengthening the antioxidant capability of PC12 cells.

### 3.6. Nrf2 Nuclear Accumulation Mediates the Neuroprotective Effect of ISL Against Radiation Injury

**Figure 6:**
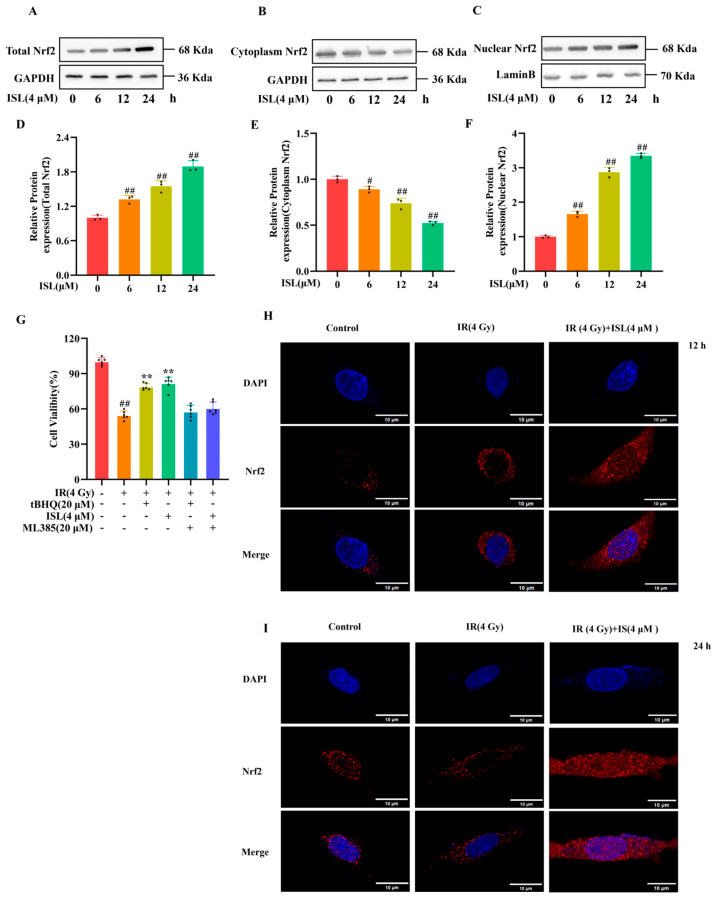
Nrf2 nuclear accumulation mediates the protective effect of ISL on irradiated neuronal cells. (**A**–**F**) Promotion of Nrf2 nuclear accumulation by ISL. PC12 cells were treated with 4 μM ISL for 0, 6, 12, and 24 h. Western blot analysis was performed to detect the protein levels of (**A**,**D**) total Nrf2, (**B**,**E**) cytoplasmic Nrf2, and (**C**,**F**) nuclear Nrf2. GAPDH and Lamin B were used as loading controls (n = 3). (**G**) The inhibitory effect of ML385 on Nrf2 can reverse the radioprotective effect of ISL against radiation-induced injury in PC12 cells (n = 6). (**H**,**I**) Effect of ISL on Nrf2 nuclear translocation in PC12 cells treated for (**H**) 12 h and (**I**) 24 h (scale bars: 10 μm). ^#^ *p* < 0.05, ^##^ *p* < 0.01 vs. the control group; ** *p* < 0.01 vs. the IR group.

To explore whether ISL activates Nrf2 and facilitates its translocation from the cytoplasm to the nucleus, Western blot and immunofluorescence assays were performed to detect Nrf2 expression in PC12 cells. As shown in Figure 6A–F, total Nrf2 protein expression was significantly upregulated in ISL-treated PC12 cells compared with the 0 h group (*p* < 0.01). Further detection of cytoplasmic and nuclear Nrf2 protein levels revealed a marked reduction in cytoplasmic Nrf2 accompanied by a notable increase in nuclear Nrf2 (*p* < 0.01), suggesting the occurrence of Nrf2 nuclear translocation. ML385, a specific inhibitor of Nrf2, was used to clarify the involvement of Nrf2 in the protective effect of ISL against radiation injury. Cell viability was markedly reduced following radiation exposure (*p* < 0.01), whereas ISL treatment effectively restored the viability of irradiated PC12 cells (*p* < 0.01). Notably, combined treatment with ISL and ML385 obviously abrogated the protective action of ISL against radiation-induced damage (Figure 6G). These results demonstrate that the neuroprotective effect of ISL is dependent on the Nrf2 signaling pathway.

Consistent with the above findings, the immunofluorescence results (Figure 6H,I) showed that Nrf2 fluorescence intensity was significantly enhanced in ISL-treated PC12 cells relative to the control group, accompanied by evident nuclear aggregation of Nrf2. This effect reached the maximum at 24 h after ISL intervention. Overall, ISL effectively promotes Nrf2 translocation and nuclear accumulation in PC12 cells.

### 3.7. ISL Improves Learning and Memory Abilities of Mice

**Figure 7:**
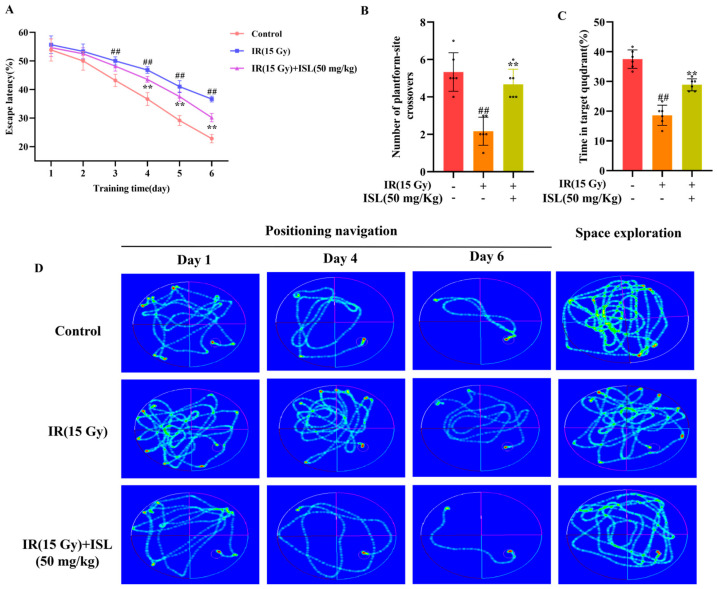
ISL ameliorates radiation-induced neuronal damage and improves learning and memory in mice. (**A**) Escape latency to the hidden platform in the place navigation trial. (**B**) Number of crossings over the original platform site in the spatial probe test. (**C**) Time spent in the target quadrant during the spatial probe test (n = 6). (**D**) Swimming path trajectories in place navigation (Days 1, 4, and 6) and spatial probe test. ^##^ *p* < 0.01 vs. the control group; ** *p* < 0.01 vs. the IR group.

The Morris water maze test was performed to evaluate the ameliorative effect of ISL on learning and memory functions in mice. As shown in Figure 7A, compared with the control group, escape latency was significantly prolonged in mice following radiation exposure. By contrast, ISL treatment markedly shortened the escape latency. In the spatial probe test, the model group spent obviously less time in the target quadrant and made fewer crossings over the original platform position relative to the control group (*p* < 0.01). Compared with the model group, ISL intervention remarkably increased both the residence time in the target quadrant and the number of platform crossings (*p* < 0.01). These findings suggest that ISL effectively ameliorates radiation-induced neuronal damage and improves learning and memory deficits in irradiated mice (Figure 7).

### 3.8. ISL Ameliorates Pathological Damage in Different Brain Regions of Mice

**Figure 8:**
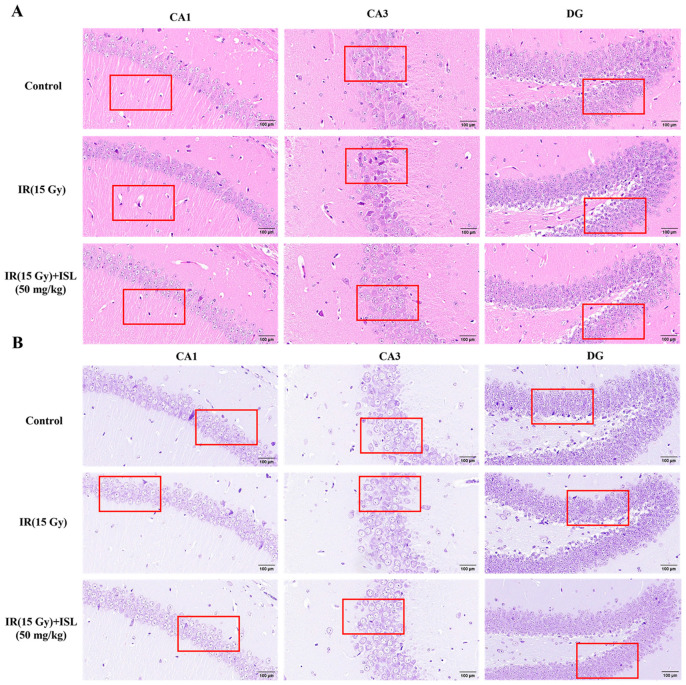
ISL ameliorates IR-induced pathological damage in mouse hippocampal tissue. (**A**) Representative images of HE staining in mouse hippocampal tissue (scale bars: 100 μm). (**B**) Representative images of Nissl staining (scale bars: 100 μm). As shown in the red box in the figure, the pathological damage in the hippocampus of the mouse brain tissue was significantly improved after ISL administration. As shown in the red box in the figure, the pathological damage in the hippocampus of the mouse brain tissue was improved after ISL administration.

HE and Nissl stainings were performed to observe histological changes in the hippocampal CA1, CA3, and DG regions of mice. The representative HE staining results are presented in Figure 8A. In the control group, hippocampal neurons were regularly and densely arranged with an intact cellular structure, clear nuclear and cytoplasmic contours, an evenly stained cytoplasm, and distinct nuclei. The perivascular spaces also maintained a normal morphology. Compared with the control group, the irradiation model group exhibited a disorganized neuronal arrangement in the CA3 region, markedly dilated perivascular spaces in the CA1 and DG regions, and extensive neuronal deformation in the CA3 region. These injured neurons showed an abnormal nuclear morphology and intense hyperchromatic staining. After ISL intervention, the neuronal arrangement in the DG region was significantly restored, and the perivascular spaces in the CA1 region were partially normalized. Moreover, the demarcation between the nuclei and cytoplasm in the hippocampus became clearer, accompanied by alleviated nuclear pyknosis and hyperchromasia. Overall, ISL markedly attenuated radiation-induced histological damage in the mouse hippocampus.

The Nissl staining results are presented in Figure 8B. In the control group, neurons in the hippocampal CA1, CA3, and DG regions were regularly arranged with an intact morphological structure, clear nuclei, and abundant well-defined Nissl bodies. Compared with the control group, the model group exhibited a disorganized neuronal distribution in the CA1 and DG regions, accompanied by neuronal swelling or shrinkage. In the CA3 region, Nissl bodies became blurred and indistinct in partial neurons, while neuronal density was markedly reduced in the DG region. After ISL intervention, neuronal pathological injuries in the hippocampal CA1 and DG regions were obviously alleviated. Neuronal arrangement became more regular, with clear nuclei and intact peripheral Nissl bodies. Moreover, the morphological structure of neurons in the CA3 region was partially restored. Overall, ISL markedly ameliorates radiation-induced histopathological abnormalities and Nissl body loss in the mouse hippocampus.

### 3.9. ISL Upregulates Nrf2 Expression in Mouse Brain Tissue

**Figure 9:**
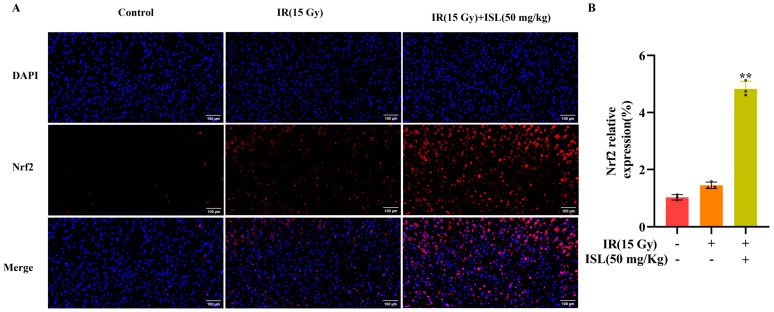
ISL upregulates Nrf2 expression in the cerebral cortex of radiation-exposed mice. (**A**) Nrf2 immunofluorescence staining of mouse brain tissue (scale bars: 100 μm). (**B**) Nrf2 fluorescence intensity statistics (n = 3). ** *p* < 0.01 vs. the IR group.

Immunofluorescence staining was performed to detect the expression of Nrf2 in the cerebral cortex of mice. As shown in Figure 9A,B, the model group exhibited a slight increase in Nrf2 protein expression compared with the control group. Notably, ISL treatment further markedly upregulated Nrf2 protein levels (*p* < 0.01), accompanied by a remarkable enhancement of red fluorescence intensity (Figure 9A). These results suggest that ISL promotes Nrf2 expression in the cerebral cortex following radiation-induced neuronal injury. Overall, ISL effectively upregulates Nrf2 expression in mouse brain tissue.

## 4. Discussion

Radiation therapy is a cornerstone treatment for brain tumors, yet it inevitably injures adjacent healthy brain tissue and triggers a spectrum of adverse side effects, including impairments in learning, memory, and attention. Despite substantial advances in radiotherapy techniques, radiation-induced brain injury remains a prevalent clinical complication that leads to progressive cognitive dysfunction of varying severity [28]. Recent studies have explored multiple therapeutic strategies for radiation-induced brain injury. In particular, emerging interventions based on natural compounds and pharmacological agents can modulate oxidative stress, antioxidant defense, inflammatory mediators, and pathological changes, exhibiting considerable potential for alleviating radiation-related brain damage [29]. Overall, although radiation-induced brain injury remains a challenging clinical complication, accumulating research advances and the development of novel therapeutic strategies have rendered this condition increasingly manageable, offering patients more intervention options and improved prognostic prospects. Accordingly, the present study aimed to explore the therapeutic efficacy and underlying mechanisms of licorice blood-absorbed constituents against radiation-induced brain injury. Our results revealed that ISL, a key bioactive constituent absorbed into the bloodstream from licorice, markedly improved the viability of radiation-injured PC12 cells and alleviated cellular damage caused by oxidative stress. In addition, oral ISL administration not only rescued learning and memory deficits in mice with radiation-induced brain injury but also attenuated radiation-triggered histological structural damage in brain tissues.

Decades of research have demonstrated that persistent oxidative stress induced by IR is critically involved in the progression of neurodegenerative injury. This process is mainly driven by the excessive generation and accumulation of ROS. It is well established that IR triggers excessive ROS generation, which serves as a pivotal mediator underlying the onset and progression of radiation-related neurodegenerative injury [30]. Furthermore, oxidative stress is a critical factor in the neurotoxicity of dopaminergic neurons, with free radicals causing substantial cellular damage [31]. Nrf2 acts as a key cellular stress sensor, triggering the expression of various antioxidant and cytoprotective genes by binding to ARE [32]. Under normal physiological conditions, Nrf2 is retained in the cytoplasm through its interaction with Keap1 and is subsequently degraded via ubiquitin-mediated proteasomal activity. However, upon exposure to radiation-induced oxidative stress, Keap1 undergoes post-translational modifications, promoting the translocation of Nrf2 into the nucleus. This process initiates ARE-dependent gene expression, enabling cells to counteract oxidative stress [33]. The genes regulated by the Nrf2-Keap1-ARE pathway include GSH-Px, GST, NQO1, SOD1, and HO-1, as well as the glutamate–cysteine ligase subunits GCLC and GCLM. Collectively, these molecules serve as core components of the endogenous antioxidant defense system [34]. Over the years, it has been well established that oxidative stress is a pivotal participant in a wide range of pathophysiological processes, particularly in the pathogenesis of neurodegenerative disorders characterized by the progressive loss of specific neuronal subpopulations. Accordingly, therapeutic strategies aimed at preventing or alleviating neuronal injury caused by radiation-associated oxidative stress have attracted substantial research attention.

**Figure 10:**
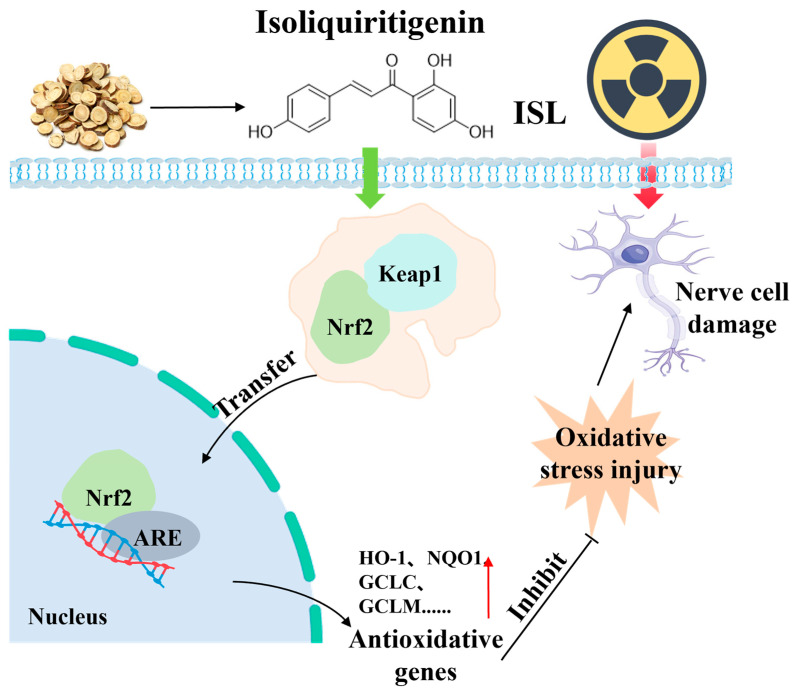
ISL can activate the Keap1-Nrf2 signaling pathway and inhibit oxidative stress to counteract radiation-induced neural injury.

We first performed a preliminary metabolic profiling of the blood-absorbed constituents of licorice and identified multiple compounds, mainly flavonoids and terpenoids. Molecular docking analysis of Keap1 revealed that flavonoids such as apigenin, naringenin, and ISL exhibited relatively high binding affinity scores for Keap1. However, apigenin, a naturally occurring flavonoid, is widely distributed in common fruits, vegetables, and spices. Major dietary sources include parsley, celery, and citrus fruits, as well as medicinal herbs such as chamomile and thyme [35]. Naringenin is mainly found in citrus fruits such as grapefruit; vegetables and fruits like tomatoes and cherries; and foods derived from medicinal plants [36]. On the contrary, ISL is widely recognized as one of the most bioactive components in licorice, and, as a characteristic flavonoid active constituent in licorice, it has a stable source and definite content [37]. Therefore, compared with apigenin and naringenin, the properties of ISL are more consistent with those of the traditional Chinese medicine licorice. In addition, studies have shown that the content of ISL in a 95% ethanol extract of licorice can reach 24 mg/kg [38]. For the above reasons, ISL was selected as the main target for subsequent experimental studies in the present research. ISL has been extensively investigated for its potent antioxidant and anti-inflammatory properties [39,40]. However, the potential of ISL in radiation protection remains underexplored and has not yet gained widespread recognition. In an ongoing effort to identify novel small-molecule regulators of the cellular redox system [7,41,42], the present study demonstrates for the first time that ISL confers prominent neuroprotection against radiation-induced injury in PC12 cells and mouse brain tissue. Mechanistically, ISL achieves this effect by activating the Nrf2 signaling pathway and upregulating the expression of phase II antioxidant and detoxification enzymes, the specific mechanism is shown in Figure 10. Our research demonstrated that radiation exposure significantly reduced the viability of PC12 cells and increased LDH release, whereas ISL treatment markedly enhanced cell viability and decreased LDH release, suggesting a protective effect of ISL against radiation-induced damage in PC12 cells. T-AOC serves as a comprehensive indicator for assessing the functionality of the antioxidant system, reflecting the levels of various antioxidant substances in the body [43]. SOD1 is a critical antioxidant enzyme in the body that catalyzes the dismutation of superoxide anion radicals into hydrogen peroxide and oxygen. Under normal physiological conditions, SOD1 effectively scavenges free radicals and helps maintain cellular redox homeostasis [44]. GCLC and GCLM serve as the catalytic and regulatory subunits, respectively, and are rate-limiting enzymes for GSH biosynthesis. Nrf2 promotes GSH biosynthesis by GCLC and GCLM expression, thereby enabling GSH to more effectively neutralize reactive oxygen species such as hydrogen peroxide within cells [45]. Our study revealed that radiation decreased the levels of T-AOC in cells, while ISL alleviated this phenomenon, suggesting its ability to enhance cellular antioxidant capacity. This may be attributed to ISL activating the intracellular antioxidant defense system, thereby alleviating oxidative stress damage induced by radiation. Subsequently, we examined the expression of Nrf2 downstream antioxidant and detoxification factors, including SOD1, HO-1, NQO1, GCLC, GCLM, Trx1, and TrxR1. The results demonstrated that ISL significantly upregulated the expression of these proteins and genes, suggesting that ISL may maintain oxidative stress balance in radiation-damaged PC12 cells by modulating the Nrf2 pathway, thereby contributing to the prevention and treatment of radiation-induced neural damage. Under normal conditions, Nrf2 remains inactive when bound to Keap1. During oxidative stress, dissociation occurs, leading to Nrf2 translocation into the nucleus and initiating the expression of a series of antioxidant genes [46]. The activation of the aforementioned antioxidant and detoxification factors is closely associated with the nuclear translocation of Nrf2. To further explore whether ISL can facilitate the translocation of Nrf2 from the cytoplasm to the nucleus, we performed additional experiments. Western blotting and immunofluorescence staining were applied to assess the expression and distribution of Nrf2 proteins in the cytoplasm and nucleus of cells. The results showed that ISL significantly increased Nrf2 expression and facilitated its translocation from the cytoplasm to the nucleus, suggesting that ISL may function as a potent Nrf2 activator and confer neuroprotection against radiation-induced damage via promoting Nrf2 nuclear translocation.

After verifying the role of ISL at the cellular level and elucidating its mechanism of action, we further examined the therapeutic effects of ISL on a mouse model of radiation-induced brain injury. The research showed that, compared with the control group, mice with radiation-induced neural damage exhibited slower movement, darker fur, and reduced body weight and food/water intake. After ISL administration, the mental state of the mice improved significantly, and their body weight and food/water intake increased. Morris water maze experiment results indicated that, compared with the control group, the escape latency of radiation-induced neuroinjury mice was significantly prolonged, while the time spent in the target quadrant and the number of platform crossings were significantly reduced, suggesting impaired learning and memory abilities. After ISL administration, the escape latency was significantly shortened, and the time spent in the target quadrant and the number of platform crossings were significantly increased, indicating that ISL could improve the learning and memory abilities of radiation-induced neuroinjury mice. HE staining and Nissl staining results revealed that ISL administration prevented the expansion of the perivascular space in the hippocampus, improved cell morphology, reduced hippocampal injury, exerted neuroprotective effects, and maintained learning and memory functions. Immunofluorescence results showed that Nrf2 expression was markedly elevated in the brain tissue of mice with radiation-induced brain injury compared with the control group, indicating that radiation induces oxidative stress and increases Nrf2 expression. After ISL administration, Nrf2 expression was significantly upregulated, suggesting that ISL promotes Nrf2 protein expression in radiation-induced neuroinjury mice. ISL exhibits neuroprotective effects and can alleviate learning and memory impairments in radiation-induced neuroinjury mice. This is related to its ability to improve hippocampal neuron structure, increase Nrf2 expression in mouse brain tissue, and activate the Keap1-Nrf2 pathway to inhibit oxidative stress.

In summary, this study analyzed and screened licorice blood components using UHPLC-Q-Exactive Orbitrap MS technology and molecular docking technology, identifying the active component ISL that activates the Keap1-Nrf2 signaling pathway. Further cellular and animal experiments explored the protective effects and mechanisms of ISL in radiation-induced neuronal injury. These findings reveal the rationale underlying the traditional application of licorice in the prevention and treatment of radiation-associated neurodamage, laying a solid theoretical foundation for the development of novel neuroprotective agents. Licorice is also rich in triterpene saponins, flavonoids, and other bioactive constituents. Whether these components possess antioxidant and neuroprotective effects, as well as their underlying molecular mechanisms, warrants further exploration. In addition, PC12 cells, a rat-derived pheochromocytoma cell line, originate from an irradiation-induced adrenal medullary tumor [47]. Owing to their functional and phenotypic similarities to neuron-like cells, they are an accessible surrogate for primary neurons. Consequently, they have been extensively employed in neuroscience research, including studies on neurotoxicity, neuroprotection, neurite outgrowth, and intracellular signaling pathways [48,49,50]. Although PC12 cells are a widely accepted neuronal model, particularly after NGF differentiation, they remain a tumor-derived cell line and may not fully reflect the physiology of mature neurons. Therefore, future studies should validate our findings using primary neuronal cultures or brain organoid slices. The above research aims to provide a theoretical basis and reference for the clinical treatment of radiation-induced neuronal injury. However, whether ISL can protect nerve cells through other mechanisms is still to be further investigated.

## 5. Conclusions

Our findings indicate that ISL, a bioactive blood component derived from licorice, may alleviate radiation-induced brain injury (RBI) by modulating oxidative stress responses via the Nrf2 signaling pathway. This study is anticipated to offer insights and references for the clinical management of radiation-induced neural injury, highlighting its potential for further development and application in RBI treatment.

## Figures and Tables

**Table 1:** Primer sequences used for qRT-PCR.

Gene	Forword Sequence	Reverse Sequence	Gene Accession Number
*HO-1*	5′-gccctggaaggagatagag-3′	5′-tagtgctgtgtggctggtgt-3′	*NM_012580.2*
*NQO1*	5′-tcaccactctactttgctccaa-3′	5′-ttttctgctcctcttgaacctc-3′	*NM_017000.3*
*Trx1*	5′-ccttctttcattccctctgtgac-3′	5′-cccaaccttttgaccctttttat-3′	*NM_053800.3*
*TrxR1*	5′-actgctcaatccacaaacagc-3′	5′-ccacggtctctaagccaatagt-3′	*NM_031614.3*
*GCLC*	5′-caaggacaagaacacaccatct-3′	5′-cagcactcaaagccataacaat-3′	*NM_012815.2*
*GCLM*	5′-ggcacaggtaaaacccaatagt-3′	5′-ttcaatgtcagggatgctttct-3′	*NM_017305.2*
*SOD1*	5′-tccctgacctgccttacgact-3′	5′-tccagcaactctcctttgggt-3′	*NM_017050.1*
*GAPDH*	5′-cagtgccagcctcgtctcat-3′	5′-aggggccatccacagtcttc-3′	*NM_017008.4*

**Table 2:** Identification of licorice components entering the bloodstream.

Nomenclature	Mass-to-Charge Ratio	Retention Time/Min	Peak Area	Ion Mode
Apigenin	433.11	3.94	16,112,482	+
Gentisic acid	153.02	4.06	11,169,978	−
18-β-Glycyrrhetinic acid	470.34	9.39	37,894,259	+
Naringenin	273.08	5.00	3,771,339.3	+
Lauramide	200.2	8.36	6,524,675.6	+
Isoliquiritigenin	255.07	5.65	5,655,037.6	+
Formononetin	267.07	6.64	2,636,039	−
Asarone	207.1	8.93	2,898,940	−
Ruspolinone	250.14	3.69	6,311,073.8	−
Glycitin	447.13	5.74	2,356,952.3	+
2,4-diacetylphloroglucinol	209.04	3.89	19,911,704	−
Coumarin	147.04	1.38	3,616,435.5	+
Eugenitin	219.07	7.42	1,751,986.5	−
Steviol	319.23	9.06	3,630,953	−

**Table 3:** Summary of molecular docking results of licorice blood components with Keap1.

Compound	Structure	Connect the Points (kcal/mol)
Apigenin	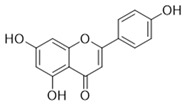	−6.934
Naringenin	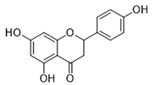	−6.762
Isoliquiritigenin	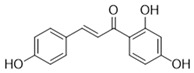	−5.93
Gentisic acid	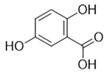	−6.025
Ruspolinone	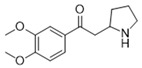	−5.907
Formononetin	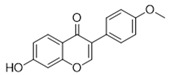	−5.864
Eugenitin	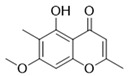	−5.722
Coumarin	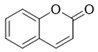	−5.654
Glycitin	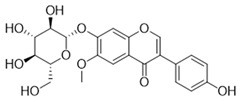	−5.497
Steviol	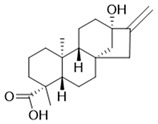	−5.294
Asarone	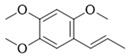	−5.097
2,4-diacetylphloroglucinol	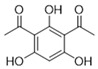	−4.831
18-β-Glycyrrhetinic acid	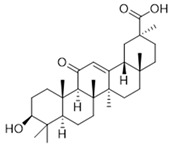	−4.579
Lauramide	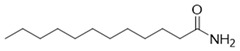	−1.137

**Table 4:** Binding free energy and energy terms.

Complex	Energy Component (kcal/mol)				
	$∆E_{\mathrm{vdw}}$ ^a^	$∆E_{\mathrm{ele}}$ ^b^	$∆G_{\mathrm{gas}}$ ^c^	$∆G_{\mathrm{solv}}$ ^d^	$∆G_{\mathrm{bind}}$ ^e^
**K** **eap1-ISL**	−26.82 ± 0.23	−46.67 ± 0.80	−73.49 ± 0.75	52.84 ± 0.59	−20.65 ± 0.24

Note: ^a^ van der Waals interaction energy ($∆E_{\mathrm{vdw}}$) in gas phase. ^b^ Electrostatic interaction energy ($∆E_{\mathrm{ele}}$) in the gas phase. ^c^ Gas-phase Gibbs free energy change ($∆G_{\mathrm{gas}}$). ^d^ Contribution of solvation energy ($∆G_{\mathrm{solv}}$). ^e^ Total binding free energy, $(∆G_{\mathrm{bind}}\;=\;∆E_{\mathrm{vdw}}\;+\;∆E_{\mathrm{ele}}\;+\;∆G_{\mathrm{pol}}\;+\;∆G_{\mathrm{nonpol}}$).

## Data Availability

The data presented in this study are available on request from the corresponding authors.

## Abbreviations

The following abbreviations are used in this manuscript:

AAPH	2,2′-Azobis(2-methylpropionamidine) dihydrochloride
ABTS	2,2′-Azinobis(3-ethylbenzothiazoline-6-sulfonic acid)
ARE	Antioxidant response element
CCK-8	Cell counting kit-8
DCFH-DA	2′,7′-Dichlorodihydrofluorescein diacetate
DPPH	2,2-Diphenyl-1-picrylhydrazyl
ELISA	Enzyme-linked immunosorbent assay
ECL	Enhanced chemiluminescence
GAPDH	Glyceraldehyde-3-phosphate dehydrogenase
GCLC	Glutamate–cysteine ligase catalytic subunit
GCLM	Glutamate–cysteine ligase modifier subunit
GSH	Glutathione
GSH-Px	Glutathione peroxidase
GST	glutathione S-transferase
HE	Hematoxylin–eosin
HO-1	Heme oxygenase-1
IR	Ionizing radiation
ISL	Isoliquiritigenin
Keap1	Kelch-like ECH-associated protein 1
LDH	Lactate dehydrogenase
Nrf2	Nuclear factor erythroid 2-related factor 2
NQO1	NAD(P)H: quinone oxidoreductase 1
OPLS-DA	Orthogonal partial least squares discriminant analysis
PC12	Pheochromocytoma cell line
PBS	Phosphate-buffered saline
PCA	Principal component analysis
qRT-PCR	Quantitative real-time polymerase chain reaction
ROS	Reactive oxygen species
RBC	Red blood cell
SOD1	Superoxide dismutase 1
T-AOC	Total antioxidant capacity
Trx1	Thioredoxin 1
TrxR1	Thioredoxin reductase 1
UHPLC-Q-Exactive Orbitrap MS	Ultra-high-performance liquid chromatography–quadrupole-exactive Orbitrap mass spectrometry
